# The Collagen Receptor uPARAP in Malignant Mesothelioma: A Potential Diagnostic Marker and Therapeutic Target

**DOI:** 10.3390/ijms222111452

**Published:** 2021-10-23

**Authors:** Pınar Çakılkaya, Rikke Raagaard Sørensen, Henrik Jessen Jürgensen, Oliver Krigslund, Henrik Gårdsvoll, Christoffer F. Nielsen, Eric Santoni-Rugiu, Niels Behrendt, Lars H. Engelholm

**Affiliations:** 1 Finsen Laboratory, Rigshospitalet/Biotech Research & Innovation Centre (BRIC), University of Copenhagen, 2200 Copenhagen, Denmark; p.cakilkaya@bric.ku.dk (P.Ç.); hjj@finsenlab.dk (H.J.J.); Oliver.Krigslund@bric.ku.dk (O.K.); gvoll@finsenlab.dk (H.G.); eric.santoni-rugiu@bric.ku.dk (E.S.-R.); niels.behrendt@finsenlab.dk (N.B.); 2 Department of Pathology, Rigshospitalet, University of Copenhagen, 2100 Copenhagen, Denmark; rikke.raagaard.soerensen@regionh.dk; 3 Adcendo ApS, c/o Copenhagen Bio Science Park (COBIS), 2100 Copenhagen, Denmark; christoffer.nielsen@adcendo.com

**Keywords:** uPARAP, Endo180, CD280, MRC2, mesothelioma, antibody-drug conjugate, ADC, immunohistochemistry, tumor microenvironment, extracellular matrix

## Abstract

Malignant mesothelioma (MM) is a highly aggressive cancer with limited therapeutic options. We have previously shown that the endocytic collagen receptor, uPARAP, is upregulated in certain cancers and can be therapeutically targeted. Public RNA expression data display uPARAP overexpression in MM. Thus, to evaluate its potential use in diagnostics and therapy, we quantified uPARAP expression by immunohistochemical H-score in formalin-fixed paraffin-embedded bioptic/surgical human tissue samples and tissue microarrays. We detected pronounced upregulation of uPARAP in the three main MM subtypes compared to non-malignant reactive mesothelial proliferations, with higher expression in sarcomatoid and biphasic than in epithelioid MM. The upregulation appeared to be independent of patients’ asbestos exposure and unaffected after chemotherapy. Using immunoblotting, we demonstrated high expression of uPARAP in MM cell lines and no expression in a non-malignant mesothelial cell line. Moreover, we showed the specific internalization of an anti-uPARAP monoclonal antibody by the MM cell lines using flow cytometry-based assays and confocal microscopy. Finally, we demonstrated the sensitivity of these cells towards sub-nanomolar concentrations of an antibody-drug conjugate formed with the uPARAP-directed antibody and a potent cytotoxin that led to efficient, uPARAP-specific eradication of the MM cells. Further studies on patient cohorts and functional preclinical models will fully reveal whether uPARAP could be exploited in diagnostics and therapeutic targeting of MM.

## 1. Introduction

MM is a highly aggressive cancer type originating from the mesothelium lining the pleura or other serosal cavities, including the peritoneum, pericardium, and tunica vaginalis testis. Pleural MM is the most common of these, representing 90% of all cases. MM is a particularly challenging disease due to its heterogeneity, often delayed diagnosis with conventional methods and inadequate response to current treatment options. Consequently, the prognosis of pleural MM remains dismal, with median overall survival after diagnosis of approximately 12 months [1]. The MM mortality rates are increasing despite the protective measures taken to regulate asbestos, the most important causative agent of this disease [2]. There are three main histopathological subtypes: epithelioid MM (EMM), sarcomatoid MM (SMM) and biphasic MM (BMM), the latter being composed of both epithelioid and sarcomatoid components. EMM is associated with a somewhat better prognosis than the two other subtypes, while SMM has the poorest outcome in patients [1]. The treatment options for unresectable MM (75% of all cases) are very limited, given the poor efficacy of the current approved standard chemotherapy with the platin-pemetrexed doublet. These insufficient options and the hitherto disappointing attempts with targeted therapies against MM signaling pathways lately urged potential immunotherapy-based strategies to be developed against this malignancy [3,4,5]. However, there remains a crucial unmet need for identifying novel and effective therapeutic targets for MM. 

For the therapeutic utilization of novel protein targets on the cell surface, an appealing treatment option would be the antibody-drug conjugate (ADC) strategy. In principle, various cytotoxic payloads can be attached to the monoclonal antibodies (mAbs) that recognize tumor-associated proteins to achieve specific elimination. Although this strategy has indeed been studied for certain potential target proteins in MM [6,7,8,9,10,11], none of these studies has led to clinically approved ADC so far [3,12]. Therefore, it is essential to identify targetable proteins with defined expression patterns correlated with mesothelioma but with restricted expression in normal tissue. 

In previous studies, the urokinase plasminogen activator receptor-associated protein (uPARAP/Endo180, in the following designated as uPARAP) has been thoroughly investigated in terms of its function in cancer, notably including collagen degradation and cancer invasion [13,14]. In addition, however, uPARAP presents several properties that are advantageous for a potential ADC target. In particular, this protein is a recycling endocytic receptor that utilizes clathrin-associated internalization and delivers its bound cargo into the endosomal/lysosomal compartments, enabling intracellular toxin release [15]. It has a unique cellular expression pattern, including upregulation in a particular group of cancers in contrast to low expression in most non-cancerous cells, thus potentially enabling targeted drug delivery with minimal side effects. So far, uPARAP has been found to be upregulated in non-epithelial cancers, including sarcomas and glioblastomas, although the receptor also has detectable expression in certain subsets of non-malignant fibroblasts and osteoblasts [16]. We recently created a uPARAP-targeted ADC and used this reagent in a leukemia xenograft model, demonstrating a high anti-tumor effect with complete cure in all of the treated mice with no distinct side effects [17]. 

To survey additional cancer types with a potentially targetable expression of uPARAP, we performed data mining of publicly available mRNA datasets. This survey pointed to MM with an exceptionally high mRNA expression of the MRC2 gene that encodes uPARAP. Herein, we show that the uPARAP protein is highly and consistently expressed in all MM subtypes, that its expression is retained after standard chemotherapy and that the protein functions as an efficient endocytic receptor on cultured MM cells. Finally, we show that these cells can be efficiently eradicated in a target-specific manner, using an ADC comprising a specific anti-uPARAP monoclonal antibody coupled to a potent cytotoxin. 

## 2. Results

### 2.1. Upregulation of uPARAP in Malignant Mesothelioma

**Figure 1:**
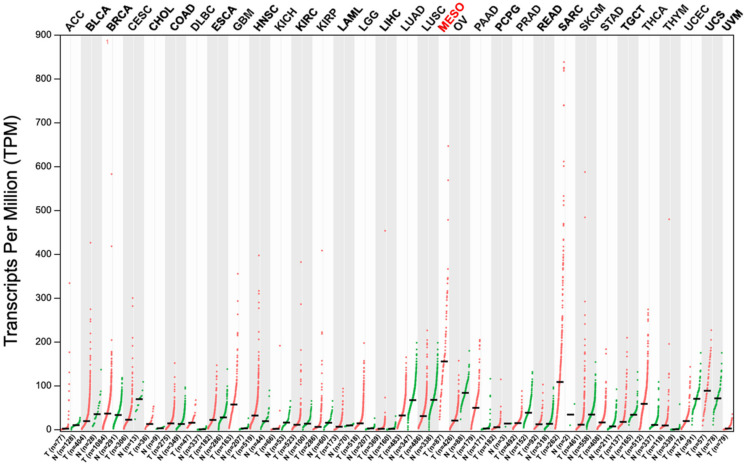
The gene expression profile of MRC2. TCGA data have been adapted from the GEPIA web server [20,21]. Dot plots (mRNA levels) across tumor samples are shown in red. MESO represents malignant mesothelioma [18]. For most tumors (although not for MM), data for their normal tissue of origin are also shown in green. The median values are indicated with bars. For the abbreviations of the analyzed cancer types, see Table S1.

To identify cancer types with a particularly high expression of uPARAP, we initially screened The Cancer Genome Atlas (TCGA) for the mRNA expression of MRC2, the uPARAP encoding gene, in various cancers. These data (originated from TCGA’s genomic profiling of MM [18] and available at [19]) pointed to modest expression levels of uPARAP in most cancers, but high mRNA levels were found in a limited number of cancer types (Figure 1). Interestingly, among all of the cancer types displayed, MM revealed the highest median level of uPARAP expression. In particular, this level was higher than that of any normal tissue. With these data as a preliminary indication, we then performed an immunohistochemical study of the uPARAP protein in MM. 

First, we retrospectively studied uPARAP protein expression on a series of formalin-fixed paraffin-embedded (FFPE) tissue sections from 28 patients diagnosed with pleural MM and its histopathological subtypes previously determined according to international guidelines [22]. For immunohistochemistry (IHC), we utilized diagnostic thoracoscopic biopsies from chemotherapy-naïve patients and surgical extended pleurectomy/decortication (P/D) specimens from patients after neoadjuvant chemotherapy and analyzed these specimen types separately. All together, these samples represented a total of 7 patients with EMM, 11 with BMM, and 10 with SMM (including the strongly collagenized variant of SMM called desmoplastic malignant mesothelioma (DMM) from 4 patients). For 12 MM patients (5 with EMM, 6 with BMM, and 1 with SMM), paired thoracoscopic biopsies and resection specimens were used for assessing the effect of chemotherapy on uPARAP expression (see underneath in this section), while only one of these two specimen types was available for the other 16 MM patients. As non-malignant controls, sections from 12 cases of reactive mesothelial proliferation (RMP) and variable fibrosis of the pleura obtained from patients operated for other intrathoracic pathologies were used; see Materials and Methods and Table S2 for the demographic description of the patients.

The tissue sections were immunostained with a specific antibody against uPARAP [23,24], and the expression of this protein was quantified by H-score as described in Materials and Methods. For histological evaluation to confirm the original diagnoses, parallel adjacent tissue sections from all these specimens were stained with standard H&E and immunostained for pan-cytokeratin, an epithelial cell marker that is expressed in all subtypes of MM [22].

**Figure 2:**
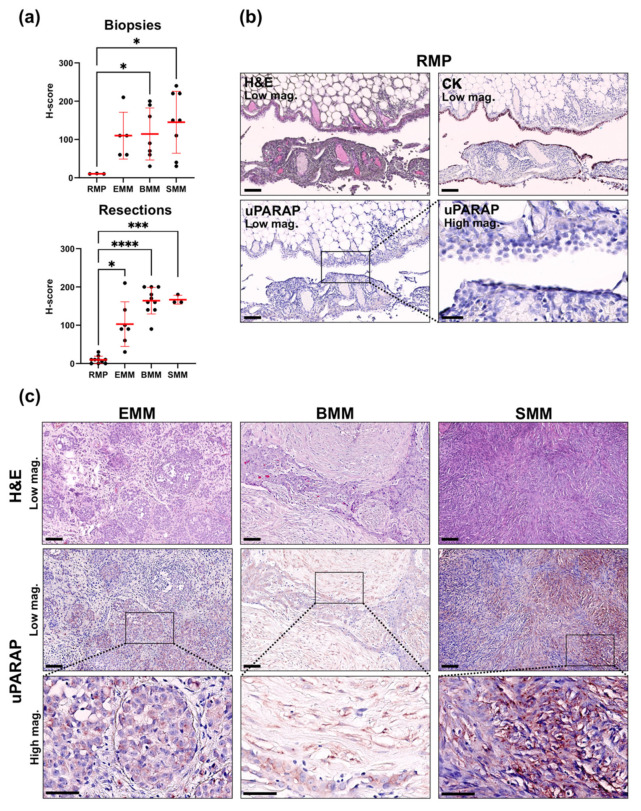
IHC analysis of uPARAP expression in tissue sections from MM and RMP. (**a**) Graphs indicate H-scores that were generated by uPARAP IHC analysis of FFPE tissue sections from biopsies taken from chemotherapy-naïve patients (upper graph: RMP (*n* = 3), EMM (*n* = 5), BMM (*n* = 7), SMM (*n* = 8)) or pleural resections from patients after neoadjuvant chemotherapy (lower graph: RMP (*n* = 10), EMM (*n* = 7), BMM (*n* = 10), SMM (*n* = 3)). Data are shown as mean ± SD for the individual MM subtypes and RMPs. Welch’s ANOVA test was used for the comparison of the groups. *: (*p* ≤ 0.05), ***: (*p* ≤ 0.0008), ****: (*p* ≤ 0.0001). (**b**) Representative examples of H&E, uPARAP and pan-cytokeratin (CK) staining performed on parallel tissue sections from a pleural RMP induced by spontaneous pneumothorax. The CK staining highlights the CK-positive layer of hyperplastic mesothelium covering the pleura affected by chronic inflammation, vessel proliferation and mild fibrosis. The *inset* in the image from the corresponding uPARAP immunostaining (left) is illustrated at higher magnification (right) to highlight the lack of uPARAP expression in the hyperplastic mesothelium and underlying pleural stroma. (**c**) Representative examples of H&E and uPARAP staining performed on parallel tissue sections from samples representing the three MM subtypes (EMM, BMM, and SMM). The insets in the images from uPARAP immunostainings taken at low magnification (middle row) are illustrated at higher magnification at the bottom. High magnification bars: 100 µm, low magnification bars: 50 µm.

**Figure 3:**
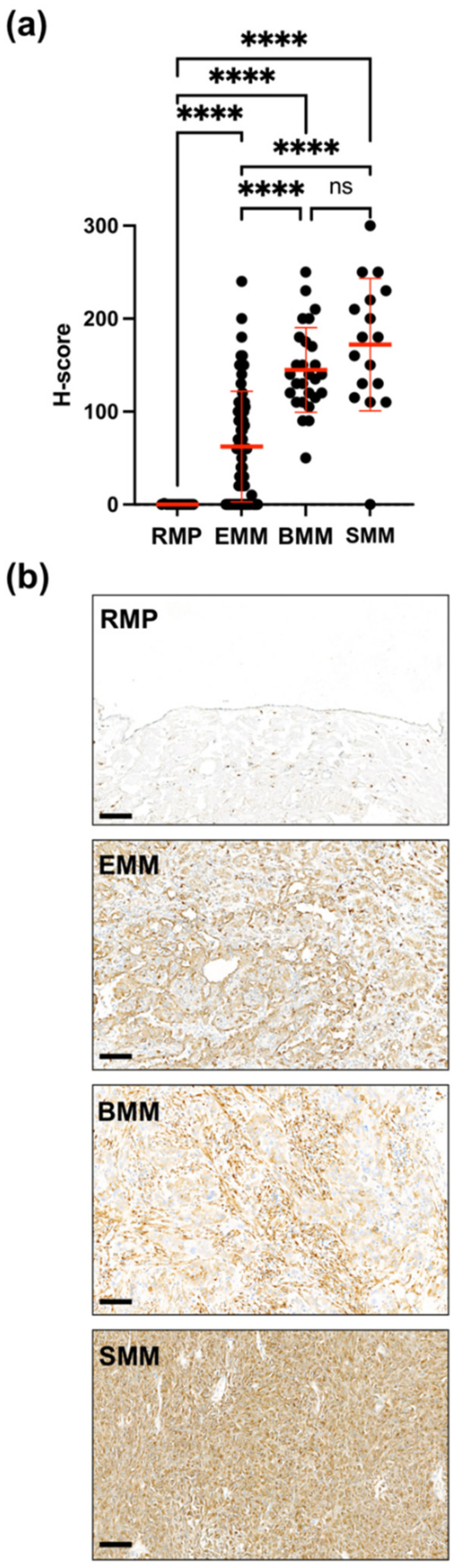
IHC analysis of uPARAP expression in TMAs from MM and RMP. (**a**) The graph indicates H-scores obtained by uPARAP IHC analysis of cores representing RMP (*n* = 13), EMM (*n* = 73), BMM (*n* = 27) and SMM (*n* = 17). Data are shown as mean ± SD for the individual MM subtypes and RMPs. Welch’s ANOVA test was used for the comparison of the groups. **** (*p* ≤ 0.0001). (**b**) Representative examples of uPARAP staining in TMA cores from RMP (no staining in the mesothelium and fibrotic stroma, except for scattered stained macrophages), EMM, BMM, and SMM tissue (all three showing diffuse positive uPARAP expression). Note the stronger stain of the sarcomatoid vs. epithelioid tumor cells in the BMM example. Scale bars: 100 µm.

The quantification results for uPARAP in the tissue sections are shown both in the biopsy material and in the resection specimens (i.e., P/D samples) (Figure 2a), with representative examples of staining shown in Figure 2b for RMP and Figure 2c for the MM subtypes. A pronounced upregulation of uPARAP was noted in the MM specimens relative to the non-malignant mesothelium in the RMPs, where the receptor was expressed at very low levels or was completely absent. The increase in expression was statistically significant for BMM and SMM, both in biopsies and resected specimens, as well as in P/D samples from EMMs (Figure 2a). In biopsies from EMMs, we observed a clear numerical upregulation (roughly a 100-fold, see Figure 2a), although this did not reach statistical significance, most likely because of the low number of included biopsies from this MM subtype. Nonetheless, this interpretation was supported by the results obtained in the resected specimens (Figure 2a) and in the tissue microarrays (TMAs) that contained a higher number of EMMs (Figure 3a).

Different cellular patterns of uPARAP staining were observed in the tumors, and in some instances, a combination of patterns was present within the same tumor sample. Three dominant staining patterns could be discerned: perinuclear, diffuse in the cytoplasm or along the cell membrane (membranous), as previously reported in other cancer types [25], indicating the localization of the uPARAP receptor in association with its recycling mechanism [15].

A comparison of the uPARAP expression levels in the individual MM subtypes was hampered by the relatively low number of samples, and no significant difference was observed between the MM subtypes in this material (Figure 2a). However, to include a more significant number of specimens, uPARAP expression was also investigated in commercially available MM TMAs. Since these specimens proved incompatible with the pre-treatment and immunostaining method that was used for the tissue sections from individual patients, a modified, automatized staining procedure was used (see Materials and Methods). The TMAs included 130 cases judged to be evaluable after the uPARAP immunostaining, including 73 EMMs, 27 BMMs, 17 SMMs, and 13 RMPs. The evaluable tumor cores in the TMAs represented cases of pleural (*n* = 42), pericardial (*n* = 10), and peritoneal (*n* = 66) MM, whereas the cores with RMPs were derived from the visceral pleura covering lung tissue (*n* = 5), mediastinal pleura (*n* = 2) and from the pericardium (*n* = 6). The analysis of these TMAs confirmed the marked upregulation of uPARAP in all of the three MM subtypes as compared to lack of expression in RMPs. However, this data set also showed significantly higher expression in SMM and BMM than in EMM (Figure 3a). Moreover, in both tissue sections and TMAs, we often detected higher expression in the sarcomatoid than the epithelioid component of BMM samples. Indeed, 19 out of 27 (70%) TMA cores and 9 out of 17 (53%) tissue sections from BMMs stained more intensely in the sarcomatoid component as compared to the epithelioid cells (see the example of BMM in Figure 3b).

The analyzed diagnostic biopsies (Figure 2a) were from thoracoscopy of therapy-naïve patients, whereas the resected samples from P/D (Figure 2b) were from EMM or BMM patients (plus one patient with SMM in whom P/D was attempted) after receiving standard neoadjuvant chemotherapy with cisplatin-pemetrexed (3 cycles for all patients). For the 12 MM cases with paired baseline biopsy and chemotherapy-exposed P/D specimens available (Table S2), the analysis of these matched samples enabled us to assess the possible effect of chemotherapy on uPARAP expression. However, we did not find any significant difference in the uPARAP H-score between these two groups of samples (*p* = 0.3149; Figure S1). We also correlated the uPARAP expression in the MM tissue sections with the asbestos exposure of the patients, using information retrieved from the corresponding clinical history. Neither this comparison revealed any significant difference in uPARAP expression between the patient groups (i.e., between patients who had been exposed and those who had not; *p* = 0.8819 and *p* = 0.9745 for the resection (P/D) and the biopsy samples, respectively; Figure S2a).

Furthermore, we investigated the correlation between the expression of uPARAP and that of the BRCA1-associated protein-1 (BAP1) gene product that had been determined during the original diagnostic workout performed on biopsies. Indeed, the loss of expression of BAP1 protein is used on diagnostic biopsies as an immunohistochemical surrogate of BAP1-mutation, one of the most frequent genetic alterations in MM, for discriminating between MM and RMP [1]. In our study, no significant correlation was identified between the BAP1 status and uPARAP expression (*p* = 0.3967; Figure S2b). 

In the TMA material, we further correlated the uPARAP expression with the tissue origin of the samples since the TMAs included cores from peritoneal and pericardial MM in addition to the pleural cases. Although mesotheliomas in different anatomical sites of the body may differ biologically and genetically [1,22], we did not find a significant difference in H-scores between different origin sites (comparing TMAs from pleural vs pericardial MM (*p* = 0.8418) or from peritoneal vs pericardial MM (*p* = 0.4305)). The comparison between TMAs from pleural and peritoneal MM showed higher expression in the former with a significant *p* value (*p* = 0.0227) (Figure S3). Besides, we observed no significant difference in uPARAP expression in the TMAs according to TNM grade (available data from Biomax) (Figure S4a) or nuclear grade (prognostic histological parameter only applicable to EMM and BMM samples [26,27]) (Figure S4b).

### 2.2. uPARAP Expression in Mesothelioma Cell Lines

**Figure 4:**
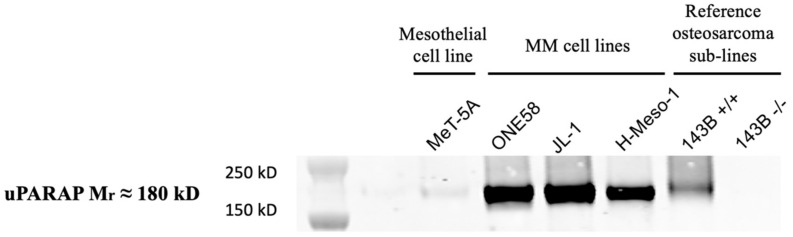
Expression of uPARAP in various cell lines. The relative expression was assessed by western blot in a non-malignant human mesothelial cell line (MeT-5A), human mesothelioma cell lines (ONE58, JL-1, H-Meso-1) and positive and negative control cells (osteosarcoma cell lines 143B +/+ and 143B -/-; see text). Bands of apparent 180 kD molecular mass indicate expression of uPARAP in all of the cancer cell lines except for the negative control. Very low expression levels were observed in MeT-5A. Comparable loading of total protein was confirmed by Coomassie^®^ blue staining of residual protein in the SDS gel, left after electroblotting (Figure S5).

Since uPARAP was found to be strongly upregulated in MM, we investigated by western blot (WB) analysis whether the expression would be retained in human MM-derived cell lines and whether these cells could be used as tools to study uPARAP-directed tumor targeting in vitro. The cells analyzed were the immortalized non-malignant mesothelial cell line, MeT-5A, the MM cell lines, ONE58, JL-1, and H-Meso-1, a subline of the osteosarcoma cell line, 143B (143B +/+; uPARAP positive control), and a negative control in the form of 143B uPARAP knock-out cells (143B -/-; see Materials and Methods). In these analyses, all of the three MM cell lines were found to be strongly uPARAP-positive, without any major difference in the expression levels (Figure 4). In contrast, just like the IHC results in tissue samples from patients with RMP, we found very low uPARAP expression in the non-malignant MeT-5A cells. The expression in the MM cell lines was at least at the same level as that in the 143B +/+ cells, whereas the 143B -/- cells proved negative in the WB experiment, documenting the specificity of detection. 

### 2.3. Internalization of a uPARAP-Directed Antibody in Malignant Mesothelioma Cells

**Figure 5:**
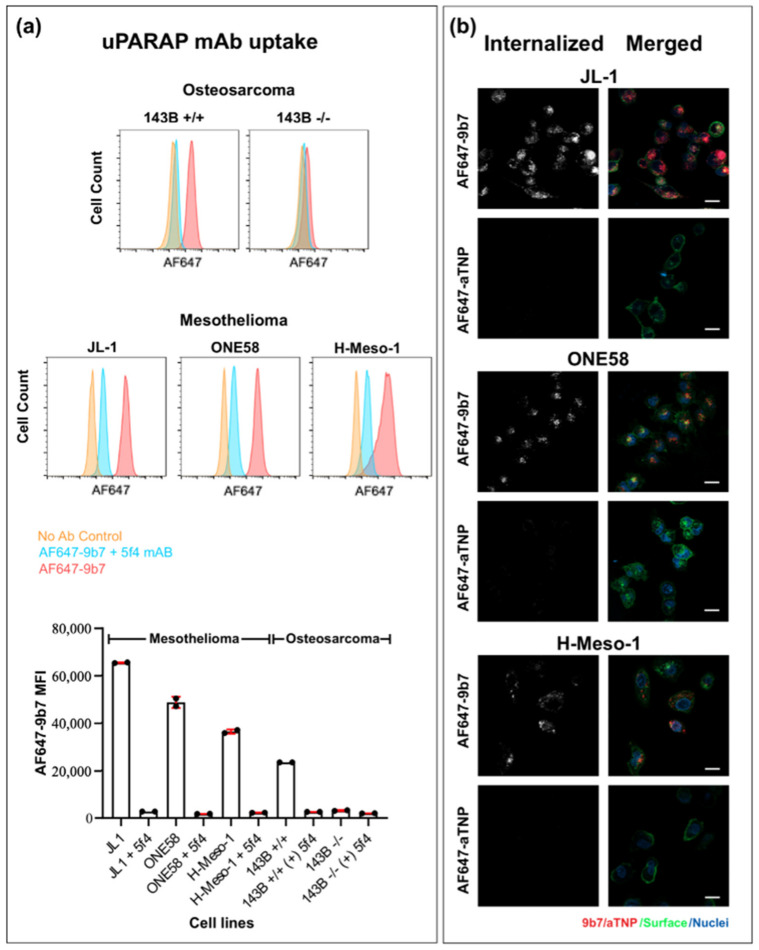
Specific uptake of anti-uPARAP mAb in MM cells. (**a**) Analysis of mAb uptake by flow cytometry. Cells were analyzed after uptake of Alexa Fluor 647 conjugated mAb 9b7 (AF647-9b7) against uPARAP. The AF647-9b7 uptake is shown as histograms (in pink) for all of the cell lines examined (MM cell lines ONE58, JL-1 and H-Meso-1 and osteosarcoma lines 143B +/+ and 143B -/-). Histograms for cells without antibody (orange) and with cells pre-incubated with competing anti-uPARAP mAb 5f4 before the addition of AF647-9b7 (light blue) are included as controls. The bar chart shows the mean fluorescence intensity of the analyzed samples (MFI ± SD). (**b**) Internalization of anti-uPARAP mAb in H-Meso-1, JL-1 and ONE58 cells, shown by confocal imaging. Cells were analyzed after uptake of AF647-9b7 against uPARAP or Alexa Fluor 647-conjugated mAb aTNP (AF647-aTNP) as a negative control. In the right panel, the merged images are displayed after incubation with AF647-9b7 (red) and AF647-aTNP (red); cells were stained with WGA Alexa Fluor 488-conjugate for surface staining (green) and Hoechst stain 33258 for visualization of the cell nuclei (blue). The internalized amount is displayed in greyscale representation (left panels). Scale bar: 20 μm.

Next, we studied the receptor-dependent endocytosis of a uPARAP-directed antibody in the MM cell lines. For this purpose, we exposed cells to a fluorescence-labeled uPARAP-specific antibody, mAb 9b7, during a 5 h incubation period to allow endocytosis. After removing the surface-bound antibody (see methods), we then analyzed the internalized fluorescence by flow cytometry (Figure 5a). All of the three MM cell lines and the 143B +/+ osteosarcoma cell line displayed a marked uptake of the fluorescent antibody, with the mean fluorescence intensity (MFI) in the MM cells even being 2 to 3-fold higher than that in the osteosarcoma cells. This internalization process was uPARAP-specific because no uptake occurred in the uPARAP-negative 143B -/- cells. As an additional specificity control, we utilized another antibody against uPARAP, mAb 5f4. In the presence of this antibody during cell culture, uPARAP becomes depleted from the cell surface, thus preventing any uPARAP-dependent endocytosis in human cells [13]. Furthermore, mAb 5f4 competes directly with mAb 9b7 for binding to uPARAP (our unpublished results). When MM cells or 143B +/+ cells were pre-incubated overnight with mAb 5f4 before the endocytosis assay, this led to at least 8-fold lower fluorescence signals compared to the non-5f4-incubated cells.

To visualize the anti-uPARAP mAb uptake and document that the antibody was internalized, we exposed MM cells to fluorescently labelled anti-uPARAP mAb 9b7 or an isotype-matched irrelevant control antibody (mAb aTNP; [28]) during incubation for 5 h. As shown in Figure 5b, a marked uptake of the uPARAP-directed mAb was observed in all of the three MM cell types (left panels), with no uptake of the negative control mAb. The anti-uPARAP fluorescence signal was indeed intracellular in all cases (right panels showing red mAb 9b7 signal and green labeling of the cell outline). 

### 2.4. uPARAP-Specific Eradication of MM Cells with a uPARAP-Directed ADC 

Having confirmed the specific internalization of anti-uPARAP mAb, we finally investigated the effect of a uPARAP-directed ADC on the viability of the MM cells. For the construction of the ADC, we coupled the anti-uPARAP mAb 9b7 to PNU-159682, a highly potent anthracyclin class cytotoxin [29], using an established linker and coupling system (see Materials and Methods). The resulting product (mAb 9b7-PEG4-VC-PAB-DMAE-PNU159682), in the following designated 9b7-PNU, was added to cells during cell culture. An equivalent ADC product was created with mAb aTNP (aTNP-PNU) and added to cells in the same manner. To determine the effect and concentration dependence of ADC treatment, cells were incubated with a serial dilution of either 9b7-ADC or aTNP-ADC in a triplicate setting for 6 days and analyzed by colorimetric cell viability assay using 3-(4,5-dimethylthiazol-2-yl)-5-(3-carboxymethoxyphenyl)-2-(4-sulfophenyl)-2H-tetrazolium (tetrazolium salt MTS; see Methods).

**Figure 6:**
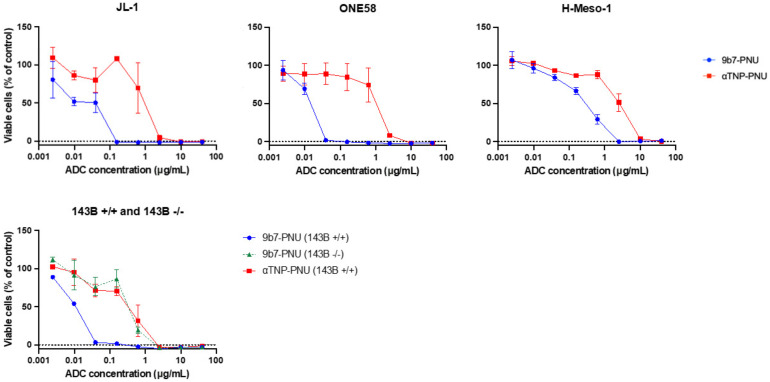
Effect of uPARAP-directed ADCs on human cancer cell lines. Graphs depict the viability of uPARAP-expressing cells after 6 days of culture in the presence of the indicated concentrations of 9b7-PNU (blue) or aTNP-PNU (red). The green dotted line shows the viability of uPARAP negative (143B -/-) cells, treated with 9b7-PNU in the same manner (receptor negative control). The percentage of viable cells is shown relative to an untreated control population. Data are presented as mean ± SD.

All of the MM cell lines, as well as the uPARAP positive 143B +/+ cells, showed a strong sensitivity to 9b7-ADC, with EC_50_ values ranging from 0.01 to 0.26 μg/mL (Table 1). This was a uPARAP-specific effect in all cases since 10 to 100-fold higher concentrations of aTNP-PNU were required to obtain an equivalent reduction in cell viability (Figure 6). Among the MM cells, the highest sensitivity to 9b7-PNU was found for the ONE58 cell line (EC_50_ = 0.014 μg/mL). This cell line also displayed the largest specificity window, with the sensitivity to 9b7-PNU being 100-fold higher than the sensitivity to aTNP-PNU. Additional proof of specificity was obtained with the osteosarcoma cells, where the 143B +/+ cells displayed a 30-fold higher sensitivity to 9b7-PNU than the 143B -/- cells (Figure 6).

## 3. Discussion

This study provides a detailed elucidation of the expression of uPARAP, a potential diagnostic marker and therapeutic target, in MM. When analyzing human specimens by IHC, a pronounced upregulation in MM relative to non-malignant (RMP) tissue was observed in all MM subtypes (Figure 2). In general, the expression of this receptor is associated with activated cells of a mesenchymal lineage [15], and it has also been demonstrated in sarcomas such as osteosarcoma [30]. In the MM patient material, the uPARAP expression in SMMs and in the sarcomatoid component of BMMs appeared to be higher than that in EMM cells (Figure 3a,b), which would be in accordance with this pattern. 

Importantly, the upregulation of uPARAP was observed both in thoracoscopic diagnostic biopsies and surgical P/D specimens (Figure 2a). The former specimens were from treatment-naïve patients, whereas the latter were from patients treated with neoadjuvant chemotherapy. The lack of significant difference when comparing these two types of patient samples (Figure S1) suggests that the expression level of the receptor is not affected by chemotherapy. Likewise, it appears that uPARAP is upregulated whether or not the patients have a history of asbestos exposure and whether or not their tumors have preserved expression of the tumor suppressor protein BAP1 (Figure S2a,b). The analysis of TMA results for the sites of origin revealed a slightly significant difference in uPARAP expression between pleural MM and peritoneal MM (Figure S3). Further studies are needed to validate and, in case, explore the biological implications of this possible difference. In contrast, we found no significant difference in uPARAP H-scores according to TNM staging or nuclear grade (Figure S4a,b). This indicates that even in the earlier stages of the disease, we might find a sufficient expression of uPARAP to target this protein with the identified levels.

From a treatment perspective, as discussed below, this means that a putative uPARAP-directed therapy might include patients of all subtypes in any stage and with any history regarding asbestos exposure. However, as would be expected, even within the individual MM subtypes, the expression of the receptor was found to be quite variable (Figure 2). This result suggests that a selection of patients for a uPARAP-directed treatment should be based on an individual evaluation of the target expression rather than a stratification based on other criteria.

A conclusive diagnosis of MM is histological and known to be challenging. In particular, the distinction between MM and benign conditions such as RMP and fibrosis in organizing pleuritis may be difficult with conventional methods. The currently employed criteria to differentiate between benign and malignant mesothelial proliferations are mainly based on a histological examination. Deep invasion into the stroma is the gold standard for establishing the diagnosis of MM so far. Still, deep invasion can be problematic to demonstrate in small biopsies, and current ancillary molecular diagnostic biomarkers (detection of CDKN2A gene deletion or loss of nuclear BAP1 protein expression suggesting the malignant nature of mesothelial cells) are not very sensitive (described in detail in [22,26]). Based on our findings, we propose that the distinct difference in the expression of uPARAP between non-malignant cases and the MM samples, if further validated, could complement the diagnostics of MM. 

Emerging molecular-targeted therapies have led to a change of focus towards personalized medicine, also in the case of MM. A number of preclinical and early clinical studies have indeed been encouraging, opening the possibility of clinical feasibility in some cases [31]. One group of drug candidates includes immunotoxins against mesothelin, a surface antigen particularly expressed on MM cells but also on normal mesothelium and other cell types. At various stages of clinical development, these drugs include anetumab ravtansine (Bayer Healthcare, BAY94-9343) [6], BMS-986148 (Bristol-Myers Squibb, New York, NY, USA) [7] and aMSLN-MMAE (Roche/Genentech, DMOT4039A, San Francisco, CA, USA) [8,9] ADCs. In addition, ADCs targeting CD228, PDL-1 and EGFR have been studied for MM treatment, carrying different payloads [10,11]. However, toxicity and difficulties in reproducing the results from phase I-II studies in larger randomized phase III trials for MM patients have appeared as limiting factors for the strategies tested so far [5]. At this point, no ADC treatment modality has been approved for MM. Therefore, the identification of a novel potential target may prove highly important.

uPARAP is a recycling endocytic receptor with lysosomal delivery of its bound cargo [15]. This property, along with the consistent upregulation of the receptor in MM as observed here, made it tempting to speculate that it might be exploited for therapy against MM by targeted drug delivery. To this end, we found that the expression of uPARAP was retained in human MM cell lines (Figure 4), which enabled us to demonstrate various essential characteristics which may support the options for targeting. 

First, we showed that MM cells were indeed capable of efficient internalization of a fluorescence-labeled, uPARAP-directed mAb. In flow cytometry experiments, all of the three MM cell lines studied displayed a marked uptake of this antibody, with the specificity of this reaction being proven by competition with a different uPARAP-specific mAb (Figure 5a). In addition, the intracellular localization of the fluorescent mAb was confirmed by confocal microscopy of single cells (Figure 5b). 

After coupling the same uPARAP-specific mAb to a secondary metabolite of nemorubicin, PNU-159682, we then showed that sub-nanomolar concentrations of the resulting ADC could efficiently eradicate cultured MM cells, although with some variation in sensitivity among the three MM cell lines examined (Figure 6). Reassuringly, this was a uPARAP-specific process since the ADC concentrations needed for cell eradication were 10- to 100-fold lower than those required with an irrelevant control ADC (cell-line dependent). These results show that efficient uPARAP-dependent drug delivery can indeed be obtained in MM cells. 

The ADC strategy for cancer treatment is a rapidly evolving field, both in research and the development of drugs that successfully reach clinical approval [32]. From the accumulating knowledge in this field, it has become clear that, for each novel ADC, a detailed optimization is needed in terms of the specific antibody and the chosen linker-toxin structure, parameters which also must be adjusted to the specific disease indication [33]. Therefore, the actual ADC format used in this work should not be seen as a proposed drug candidate for MM but rather a tool to facilitate the proof of concept. For further studies along this line, optimized ADC candidates will have to be studied in refined MM mouse models in vivo, preferably including patient-derived xenograft models [34].

Altogether, our study has pointed to uPARAP being a promising focus for further studies in MM, both as a novel molecular marker with putative diagnostic value and as a potential target for treatment. If these perspectives prove obtainable, this will fulfill an unmet need of extraordinary importance. 

## 4. Materials and Methods

### 4.1. Cell Lines

The following human cell lines were purchased from the indicated sources: MM cell line H-Meso-1 (CLS, Cat# 300186/p482_H-Meso-1, RRID: CVCL_5759); MM cell line JL-1 (DSMZ, Cat# ACC-596, RRID: CVCL_2080); MM cell line ONE58 (Sigma-Aldrich, Gillingham, UK, ECACC Cat# 10092313, RRID: CVCL_2671), and the non-malignant mesothelial cell line MeT-5A (LGC Standards, Wesel, Germany; ATCC, Cat# CRL-9444, RRID: CVCL_3749). These cells were cultured following the recommendations from the suppliers. The osteosarcoma cell line, 143B-luc2/tom-1A6, was derived from human wildtype 143B cells (ATCC Cat# CRL-8303, RRID: CVCL_2270) as described [35]. Since these cells were used as a positive control for uPARAP expression, the short designation 143B +/+ is used for the 143B-luc2/tom-1A6 cell line in this paper. The uPARAP knock-out cell line, 143B -/-, was derived from the 143B +/+ cell line by CRISPR/Cas-9 technique, using a guide RNA targeting exon 2 of the human MRC2 gene and with design and overall editing conditions as previously described [17,35]. These cells, used as a negative control for uPARAP expression, are uPARAP-deficient due to a homozygous 4 nt insertion in exon 2, which encodes the first structural domain of uPARAP. 143B +/+ and -/- cells were cultured in MEM GlutaMAX (Gibco, Life Technologies, Paisley, UK) supplemented with 1% MEM non-essential amino acids (Life Technologies Europe B.V, Bleiswijk, The Netherlands), 10% FBS (GE Healthcare Life Sciences, South Logan, UT, USA) and 1% penicillin-streptomycin (Life Technologies Europe B.V, Bleiswijk, The Netherlands).

### 4.2. Antibodies, Fluorescence Labeling and Preparation of ADCs

All mAbs used in this study belong to the murine IgG1 subtype. uPARAP-directed mAbs 2h9 and 5f4 were produced as previously described [23,24,36], using standard hybridoma technique, along with the anti-uPARAP mAb 9b7, which was obtained in the same fusion and cloning program. mAb against trinitrophenol (aTNP) has been described previously [28]. For flow cytometry and confocal microscopy, mAbs 9b7 and aTNP were labeled with Alexa Fluor 647 using a protein labeling kit (A20173, Thermo-Fisher Scientific) described by the manufacturer. The 9b7 and aTNP ADCs, formed with the cytotoxin PNU-159682 [29], were produced by cysteine-directed coupling, using a pre-formed maleimide (MA)-containing linker-toxin reagent, MA-PEG4-VC-PAB-DMAE-PNU159682 (SET0211, Levena Biopharma, San Diego, CA, USA). Coupling to the mAbs was performed as previously described [17] with minor modifications. Briefly, mAbs were subjected to a partial reduction of disulfides by treatment with 10 mM dithiothreitol (DTT) in 50 mM NaBorate 50 mM NaCl buffer (pH 8.0) for 15 min at 37 °C. Then, the partially reduced antibodies were buffer-shifted into 1 mM EDTA in PBS using 30 kDa spin filters (UFC903024, Millipore^®^, Burlington, MA, USA) and adjusted to a 2 mg/mL of IgG concentration during conjugation. The linker-toxin reagent was dissolved in water-free DMSO and added to the partially reduced antibodies at a 5-times molar excess. The reaction mixture was incubated for 2 h at 37 °C. ADCs were then isolated using PD-10 desalting columns (GE Healthcare Life Sciences) to remove the excess linker-toxin. The ADC concentration was determined by the UV/Vis program in the QIAxpert system (QIAGEN). For a qualitative evaluation of the preparations, the conjugation of the antibodies was visualized by decreased migration in SDS-PAGE of reduced samples compared with the unconjugated antibody on coomassie-stained gels, and UV/VIS absorbance spectra were recorded to ensure equal spectral properties for samples to be compared. For a quantitative determination of the molar drug-to-antibody ratio (DAR), absorbance values were determined at 280 nm and 487 nm, respectively, after which the DARs were calculated based on separately determined absorption coefficients for free antibody and free linker-toxin reagent, respectively, at the same wavelengths. The molar DARs obtained were 2.6 and 2.3 for 9b7-ADC and aTNP-ADC, respectively. The resulting shift in electrophoretic mobility of the mABs is shown in Figure S6.

### 4.3. Tissue Samples

The immunohistochemical study was performed on 53 FFPE archival tissue specimens (12 containing EMM, 17 BMM, 11 SMM, and 13 RMPs) obtained from 28 MM patients and 12 control patients with RMP who had been operated for intrathoracic pathologies not related to MM. The demographic and clinical data of the patients included in the study are listed in Table S2. The MM specimens had been obtained from diagnostic thoracoscopic pleural biopsies, and surgical extended pleurectomy/decortication (P/D) performed at the Department of Thoracic Surgery and analyzed at the Department of Pathology of Rigshospitalet, Copenhagen University Hospital, Denmark in 2017–2020. All patients pleurectomized had EMM or BMM (except one with SMM, in whom P/D was attempted because of favorable clinical condition) and had received 3 cycles of standard neoadjuvant chemotherapy with cisplatin-pemetrexed doublet. For each MM specimen, tissue sections with a tumor cell content > 50% were used. The original MM diagnosis and histological subtype of each specimen were confirmed by two thoracic pathologists (ESR and RRS), using current international guidelines [22,37]. The immunohistochemical results obtained from tissue sections were supplemented with results from MM TMAs (MS1001a, MS801b, MS481d, MS471; US Biomax, Inc., Rockville, MD, USA). These TMAs contained cores of 1.5 mm diameter in duplicates (MS1001a, MS801b, MS481d) or in one copy (MS471) from a total of 156 cases. Images of H&E staining for all the cores in the TMAs were available online at https://www.biomax.us/tissue-arrays (accessed on 12 June 2021) for histological evaluation of these samples. After immunostaining, 23 cases were excluded because of artefact (fallen-off or folded cores; no tumor tissue left in the core), so that eventually the evaluable cases were 130 comprising 73 EMMs, 27 BMMs, 17 SMMs, and 13 RMPs. Of these evaluable MMs, 66 were peritoneal, 42 pleural, and 10 were pericardial. For 77 of the MM cases, the corresponding TNM classification was provided online by Biomax. In each EMM or BMM sample (TMA cores), we scored on the corresponding H&E-stained sections the nuclear grade, which is a recognized prognostic parameter for these two MM subtypes that takes nuclear pleomorphism and mitotic count score into account [27,38].

### 4.4. Histology and Immunohistochemistry

For histological examination, 3.5 µm tissue sections were subjected to H&E staining (Histolab Products AB, Gothenburg, Sweden). Parallel sections were immunostained for uPARAP and broad-spectrum cytokeratin AE1/AE3, respectively, as follows: sections were heated for 90 min at 60 °C, followed by deparaffinization and rehydration. Antigen retrieval was then performed by treatment with 5 μg/mL proteinase K at 37 °C for 15 min. Next, the sections were washed and incubated for 15 min in a 1% H_2_O_2_ solution in Milli-Q water. Primary antibodies, mouse mAb 2h9 [23] against uPARAP and mAb mixture against pan-cytokeratin (AE1/AE3; M 3515, Dako), were diluted in antibody diluent (Dako) at 20 μg/mL and 1 μg/mL concentration, respectively. After washing, the sections were then incubated with the primary antibody solutions overnight at 4 °C. After renewed washing with TBS-T buffer, sections were incubated for 45 min at room temperature with the detection system for mouse antibodies (Envision mouse, Dako). NovaRed peroxidase substrate kit (Vector Laboratories, Inc., Burlingame, CA, USA) was then added according to manufacturer’s instructions, with incubation on sections for 9 min. Finally, sections were counterstain Mayer’s hematoxylin for 30 s. Sections incubated with antibody diluent without primary antibody were used as negative controls for the method. The stained sections were scanned by NanoZoomer-XR Digital slide scanner C12000-01(Hamamatsu) and analyzed with NDP.view2 Plus software. A modified pre-treatment and immunostaining method was used for TMA material since the TMAs proved incompatible with the method used above. For these specimens, TMAs were stained with an automatized staining procedure on a Ventana Discovery Ultra autostainer using HIER buffer protocol CC2 with 1:20,000 OTI9G4 antibody against uPARAP (TA811858, OriGene Technologies, Inc., Rockville, MD, USA) according to the manufacturer’s instructions. OmniMap-Ms-HRP and DAP substrate was used for detection.

### 4.5. Evaluation of Staining

Two experienced pathologists (ESR and RRS) evaluated the scan images in a blinded and independent manner, reaching consensus in discrepant cases. The immunostaining of uPARAP in whole tissue sections and TMAs was assessed by H-score [% of cells stained at intensity 1 (weak) × 1] + [% of cells stained at intensity 2 (moderate) × 2] + [% of cells stained at intensity 3 (strong) × 3], measuring the percentage of the area exhibiting the different intensities at 200× magnification (final score from 0 to 300), as previously described [39,40,41]. 

### 4.6. Western Blot

Cells were lysed in lysis buffer (1% Triton X-100, 50 mM Tris/HCl, and 100 mM NaCl; pH 7.4) containing protease inhibitor cocktail III (1:200; Sigma-Aldrich). After lysis at 0 °C for 20 min, lysates were clarified by high-speed centrifugation (20,000× *g* for 15 min at 4 °C). Protein concentrations in lysates were determined using Pierce™ BCA protein assay kit (Thermo-Fisher Scientific, Rockford, IL, USA). Samples of 6 μg protein from each lysate were subjected to non-reducing sample pre-treatment by boiling in NuPAGE™ LDS sample buffer (Invitrogen, Waltham, MA, USA) and electrophorezed on NuPAGE™ 4–12% gradient Bis-Tris gels (Invitrogen). Gels were electroblotted onto PVDF membranes using iBlot^®^ Dry Blotting system (Ethrog Biotechnologies, Invitrogen) for 13 min. PVDF membranes were exposed to 2% BSA blocking solution and subsequently incubated overnight at 4 °C with 1 μg/mL anti-uPARAP mAb 2h9 solution in 0.1% Tween 20 in PBS on a shaking platform. Probing was then done with goat anti-mouse Alexa Fluor Plus 800 IgG secondary antibody (1:6000; Thermo-Fisher Scientific, Rockford, IL, USA) after 45 min incubation at room temperature. Blots were imaged with Odyssey CLx fluorescence imaging system (LI-COR Biosciences, Lincoln, NE, USA) using Image Studio™ Software (version 5.2). As the loading control, 6 μg of total protein from each lysate was run by SDS-PAGE in parallel, after which the gel was stained using Coomassie brilliant blue. 

### 4.7. Confocal Microscopy

MM cells were seeded onto the cover glass of uncoated 35 mm glass-bottom culture dishes (P35G-0-14-C, MatTek In Vitro Life Science Laboratories, Bratislava, Slovakia) using 5000 cells per preparation. After overnight culture in respective complete culture media at 37 °C with 5% CO_2_, the media were exchanged with media supplemented with 2 μg/mL Alexa Fluor 647-labeled 9b7 antibody. For control preparations, 2 μg/mL Alexa Fluor 647-labeled aTNP was used. The cells were then cultured in the same conditions for an additional 5 h to allow antibody uptake. Next, cell surface staining was performed using a 1:1000 dilution of Wheat Germ Agglutinin (WGA) Alexa Fluor 488 conjugate (Thermo-Fisher Scientific, Rockford, IL, USA), and cell nuclei were counterstained with 1:1000 Hoechst stain 33258 (H3569, Thermo-Fisher Scientific). Examination of cells and acquisition of images were done using a Zeiss LSM 800 confocal microscope.

### 4.8. Flow Cytometry

For flow cytometry, cells were seeded in the wells of a 24-well plate in duplicates, in their respective media at a density of 10^5^/well, and cultured overnight. Fluorescence-labeled mAbs were then added to cells in the respective growth media at a final concentration of 1 μg/mL, followed by incubation for a further 5 h at 37 °C to allow endocytosis. Cells were then detached using 0.25% trypsin-EDTA supplemented with 50 μg/mL proteinase K. Next, cells were centrifuged at 500× *g* for 2 min at 4 °C. The pellet was resuspended in 2% FBS in PBS. In a parallel set of wells, cells were subjected to uPARAP downregulation and binding competition, using the anti-uPARAP mAb 5f4 [13]. For these wells, cells were pre-incubated overnight with the growth medium including 20 μg/mL mAb 5f4 at 37 °C. The exact concentration of mAb 5f4 was added to the assay medium during incubation with fluorescently labeled mAbs, which was otherwise performed as above. Finally, approx. 10,000 cells were analyzed on a BD Accuri™ C6 flow cytometer system (FL4 laser, 675/25 filter). For each cell line, intact, single cells were identified using Forward and Side Scatter parameters, effectively eliminating cell debris and cellular aggregates before assessing internalized fluorescence. For the calculation of the internalized fluorescence, values obtained for control samples (no addition of fluorescent material) were subtracted from the mean fluorescence values of the test samples. Data were analyzed using FlowJo software (TreeStar, Ashland, OR, USA). 

### 4.9. Assay for Cellular Sensitivity to ADCs

Cellular sensitivity to ADCs was analyzed using the MTS viability assay [17]. Cells were seeded in 100 μL of complete growth medium in tissue culture treated 96-well plates (Costar) at a density of 3000 cells/well, followed by cell culture overnight. For each ADC, a (1:4) dilution series was prepared in the complete fresh medium. In each well, the cell culture medium was then replaced with 100 μL of ADC-containing medium, using triplicate samples for each ADC concentration. Cells were then cultured for 6 days. Finally, 15 μL of CellTiter 96^®^ AQ_ueous_ One solution reagent (Promega Biotech AB, Nacka, Sweden) was added, followed by incubation for 1 h. The plates were then read in a plate reader at 490 nm and 630 nm for background subtraction. The relative cell viability was calculated by normalization to non-ADC treated control wells. EC_50_ values (μg/mL) were calculated by GraphPad Prism software, version 9.1.2. (225) (GraphPad Software Inc., La Jolla, CA, USA).

### 4.10. Statistical Analysis

Results were analyzed with GraphPad Prism. *p* values were obtained using Welch’s ANOVA test corrected by Dunnett T3. For the two-group comparisons, Welch’s *t*-test and ratio paired *t*-test have been utilized. Differences with *p* values ≤ 0.05 were considered statistically significant.

## Figures and Tables

**Table 1:** EC_50_ values for cells cultured with 9b7 or aTNP-PNU ADCs. CI, confidence interval; EC_50_, the concentration required to achieve a 50% reduction of cell viability (μg/mL).

ADC	ONE58, EC_50_ (95% CI)	JL-1, EC_50_ (95% CI)	H-Meso-1, EC_50_ (95% CI)	143B +/+, EC_50_ (95% CI)	143B -/-, EC_50_ (95% CI)
9b7-PNU	0.014 (0.011–0.016)	0.055 (0.021–0.090)	0.26 (0.174–0.339)	0.012 (0.011–0.013)	0.40 (0.233–0.558)
aTNP-PNU	1.5 (0.749–2.212)	1.1 (0.587–1.648)	2.6 (1.842–3.370)	0.33 (0.129–0.528)	-

## Data Availability

All datasets generated during and/or analyzed during the current study are available from the corresponding author on reasonable request within the legal use of confidential data.

## Supplementary Materials

The following are available online at www.mdpi.com/article/10.3390/ijms222111452/s1.
