# Supplementary material for: The Collagen Receptor uPARAP in Malignant Mesothelioma: A Potential Diagnostic Marker and Therapeutic Target
Source: Int J Mol Sci. 2021 Oct 23;22(21):11452. doi: 10.3390/ijms222111452 (PMC8583732; doi:10.3390/ijms222111452)
Supplement: Supplementary file 1 [file ijms-22-11452-s001.zip › ijms-1414792-supplementary.pdf]

Table S1. Abbreviations for TCGA cancer type.

TCGA	Detail	TCGA	Detail
ACC	Adrenocortical carcinoma	LUSC	Lung squamous cell carcinoma
BLCA	Bladder Urothelial Carcinoma	MESO	Mesothelioma
BRCA	Breast invasive carcinoma	OV	Ovarian serous cystadenocarcinoma
CESC	Cervical squamous cell carcinoma and endocervical adenocarcinoma	PAAD	Pancreatic adenocarcinoma
CHOL	Cholangio carcinoma	PCPG	Pheochromocytoma and Paraganglioma
COAD	Colon adenocarcinoma	PRAD	Prostate adenocarcinoma
DLBC	Lymphoid Neoplasm Diffuse Large B-cell Lymphoma	READ	Rectum adenocarcinoma
ESCA	Esophageal carcinoma	SARC	Sarcoma
GBM	Glioblastoma multiforme	SKCM	Skin Cutaneous Melanoma
HNSC	Head and Neck squamous cell carcinoma	STAD	Stomach adenocarcinoma
KICH	Kidney Chromophobe	TGCT	Testicular Germ Cell Tumors
KIRC	Kidney renal clear cell carcinoma	THCA	Thyroid carcinoma
KIRP	Kidney renal papillary cell carcinoma	THYM	Thymoma
LAML	Acute Myeloid Leukemia	UCEC	Uterine Corpus Endometrial Carcinoma
LGG	Brain Lower Grade Glioma	UCS	Uterine Carcinosarcoma
LIHC	Liver hepatocellular carcinoma	UVM	Uveal Melanoma
LUAD	Lung adenocarcinoma		

Table S2. Demographic description of the patients with MM or RMP.

Patient	Age-Sex	Diagnosis on the pleural specimen (cause of resection if RMP)	Surgery type	Asbestos Exposure	BAP1 IHC	TNM
1	44M	RMP (spontaneous pneumothorax)	resection from lung	-	-	-
2a	34F	RMP (spontaneous pneumothorax)	resection from lung	-	-	-
2b	34F	RMP (fibrotic pleuritis)	pleural resection	-	-	-
3	55M	RMP (lung fibrosis, UIP)	lung cryobiopsy	-	-	-
4	68F	RMP (chronic allergic alveolitis)	lung cryobiopsy	-	-	-
5	36M	RMP (spontaneous pneumothorax)	resection from lung	-	-	-
6	27M	RMP (spontaneous pneumothorax)	resection from lung	-	-	-
7	58M	RMP (aorta aneurism)	aorta resection	-	-	-
8	54M	RMP (lung transplantation)	lung explant	-	-	-
9	71 M	RMP (lung fibrosis, UIP)	surgical biopsy	-	-	-
10	29M	RMP (spontaneous pneumothorax)	resection from lung	-	-	-
11	77M	RMP (lung cancer)	pulmonary lobectomy	-	-	-
12	70M	RMP (lung cancer)	pulmonary lobectomy	-	-	-
13a	65M	Epithelioid MM	thoracoscopic biopsy	Yes	Positive	-
13b	65M	Epithelioid MM	P/D	Yes	Positive	T3N0M0
14a	58M	Epithelioid MM	thoracoscopic biopsy	No	Negative	-
14b	59M	Epithelioid MM	P/D	No	Negative	T3N0M0
15a	65M	Epithelioid MM	thoracoscopic biopsy	Yes	Positive	-
15b	65M	Epithelioid MM	P/D	Yes	Positive	T3N2M0
16a	71M	Epithelioid MM	thoracoscopic biopsy	Yes	Positive	-
16b	71M	Epithelioid MM	P/D	Yes	Positive	T3N1M0
17a	73F	Epithelioid MM	thoracoscopic biopsy	No	Negative	-
17b	73F	Epithelioid MM	P/D	No	Negative	T3N0M0
18	60M	Epithelioid MM	P/D	Yes	Negative	T2N0M0
19	65M	Epithelioid MM	P/D	Yes	Positive	T3N0M0
20a	71M	Biphasic MM	thoracoscopic biopsy	No	Positive	-

20b	71M	Biphasic MM	P/D	No	Positive	T3N0M0
21a	64M	Biphasic MM	thoracoscopic biopsy	Yes	Positive	-
21b	64M	Biphasic MM	P/D	Yes	Positive	T3N2M0
22a	67F	Biphasic MM	thoracoscopic biopsy	Yes	Negative	-
22b	67F	Biphasic MM	P/D	Yes	Negative	T3N0M0
23a	57M	Biphasic MM	thoracoscopic biopsy	No	Positive	-
23b	57M	Biphasic MM	P/D	No	Positive	T3N0M0
24a	67M	Biphasic MM	thoracoscopic biopsy	No	Negative	-
24b	67M	Biphasic MM	P/D	No	Negative	T3N2M0
25a	63M	Biphasic MM	thoracoscopic biopsy	Yes	Negative	-
25b	63M	Biphasic MM	P/D	Yes	Negative	T3NM0
26	72M	Biphasic MM	thoracoscopic biopsy	NA	Positive	-
27	70M	Biphasic MM	P/D	Yes	NA	T3N0M0
28	77M	Biphasic MM	P/D	Yes	Positive	T3N0M0
29	61M	Biphasic MM	P/D	Yes	Negative	T3N0M0
30	77M	Biphasic MM	P/D	Yes	Positive	T2N0M0
31	71M	Sarcomatoid MM	thoracoscopic biopsy	No	Negative	-
32	69M	Sarcomatoid MM	peritoneal resection	NA	Positive	-
33	77M	Sarcomatoid MM	thoracoscopic biopsy	Yes	Positive	-
34	72F	Sarcomatoid MM	thoracoscopic biopsy	NA	Positive	-
35	68M	Sarcomatoid MM	thoracoscopic biopsy	Yes	Positive	-
36a	70F	Sarcomatoid MM	thoracoscopic biopsy	No	Positive	-
36b	70F	Sarcomatoid MM	P/D	No	Positive	-
37	40M	Sarcomatoid MM	thoracoscopic biopsy	Yes	Positive	-
38	77M	Sarcomatoid MM	P/D	Yes	Positive	T3N0M0
39	73M	Sarcomatoid MM	thoracoscopic biopsy	Yes	Positive	-
40	71M	Sarcomatoid MM	thoracoscopic biopsy	No	Negative	-

***Abbreviations**: MM: malignant mesothelioma; RMP: reactive mesothelial proliferation; IHC: immunohistochemistry; UIP: usual interstitial pneumonia; P/D: pleurectomy/decortication; NA: not available.
Letters a and b represent samples from the same patient.

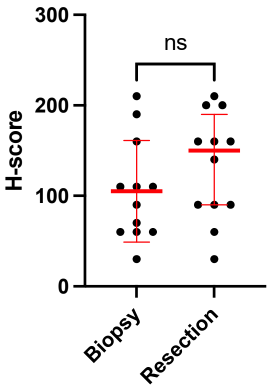

Figure S1. The H-scores of uPARAP expression in diagnostic biopsy vs. resection (P/D) samples. Biopsies (n=12) were taken at baseline from chemotherapy-naïve patients, whereas resection samples (n=12) were from matched patients after 3 cycles of neoadjuvant standard chemotherapy with cisplatin/pemetrexed doublet. The median H-scores revealed no significant effect of chemotherapy on the uPARAP expression (ratio paired t test; p= 0.3149). Data represent the mean values ± SD.

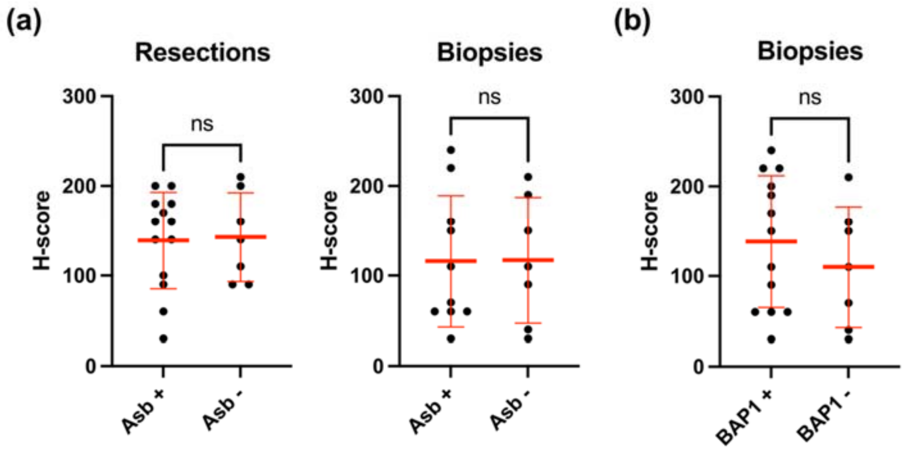

Figure S2. The uPARAP H-scores according to asbestos exposure and BAP1 expression in MM samples. Data are shown as mean ±SD. **(a)** The uPARAP H-scores according to asbestos exposure for (*left*) resection samples (Asb+ n=13; Asb- n=7), p=0.8819 and (*right*) for biopsy samples (Asb+ n=10; Asb- n=7), p=0.9745. **(b)** The uPARAP H-scores according to BAP1 expression for biopsy samples (BAP1+ n=13; BAP1- n=7), p=0.3967. Comparison was done using Welch's t-test.

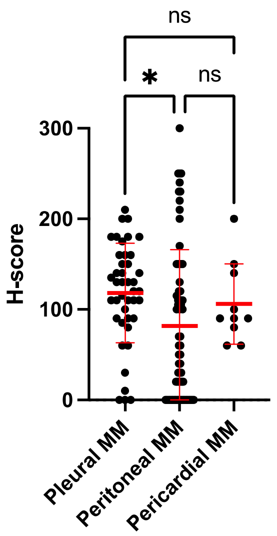

Figure S3. The uPARAP H-scores in MM TMA cores from different origin sites. The H-scores in tissue sections from pleural (n=42), peritoneal (n=66), and pericardial (n=10), MM were analyzed by Welch ANOVA test: pleural vs. peritoneal p=0.0227, pleural vs. pericardial p=0.8418, peritoneal vs. pericardial p=0.4305. Data shown as mean ±SD.

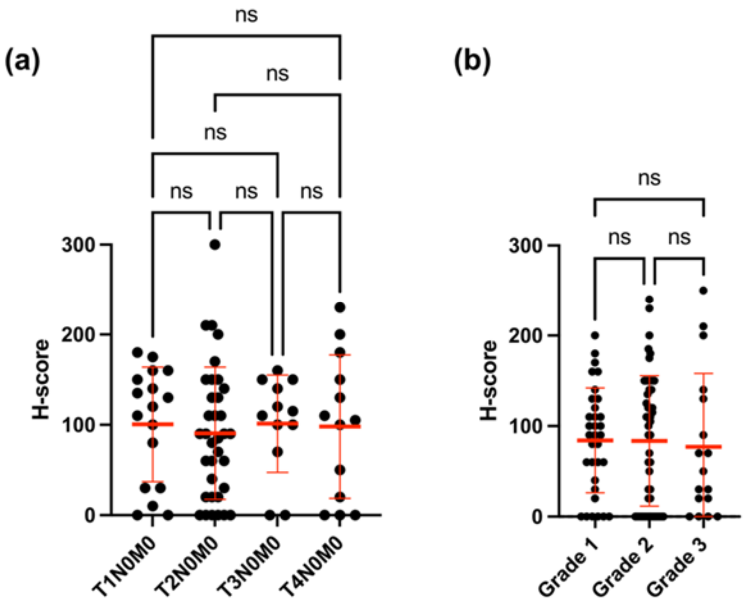

Figure S4. The uPARAP H-scores in MM TMA cores according to **(a)** TNM grade, T1N0M0 (n=17), T2N0M0 (n=35), T3N0M0 (n=12), and T4N0M0 (n=13) and **(b)** nuclear grade, which is determined for BMM and EMM tissue; Grade 1 (n=33), Grade 2 (n=50), Grade 3 (n=17). No significant difference has been found, (for ns: p>0.9999). Data are analyzed by Welch´s ANOVA test, bars shown as mean ±SD.

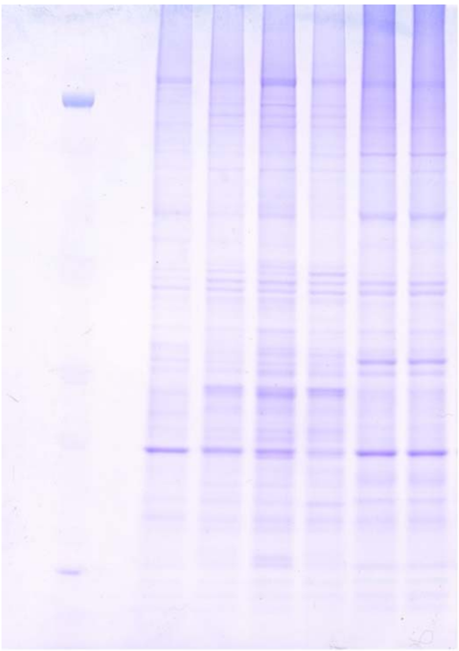

Figure S5. Coomassie staining as a loading control for the western blot experiment. After electroblotting, the residual protein left in the gel was stained with Coomassie® blue dye to confirm comparable loading.

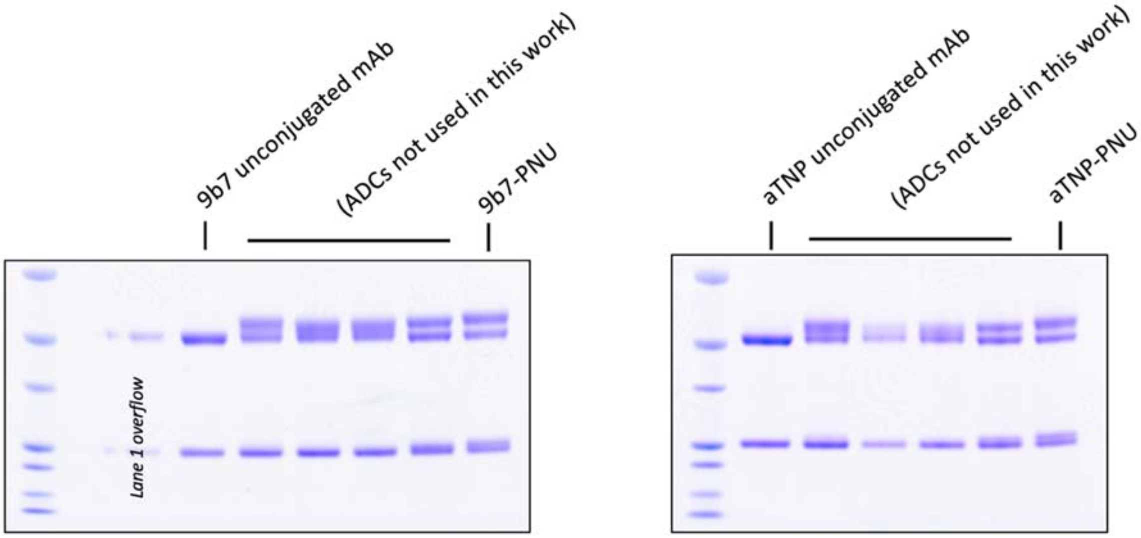

Figure S6. Covalent coupling of cytotoxic payload to mAbs, demonstrated by SDS-PAGE. Electrophoresis was performed after reducing sample pre-treatment, showing the heavy and the light chains of IgG, respectively, before and after coupling. The ADC preparations used in this work (indicated) were analysed along with other preparations.
